# Fluorescent Rhein-Loaded Liposomes for In Vivo Biodistribution Study

**DOI:** 10.3390/pharmaceutics17030307

**Published:** 2025-02-27

**Authors:** Silviu Iulian Filipiuc, Natalia Simionescu, Gabriela Dumitrița Stanciu, Adina Coroaba, Narcisa Laura Marangoci, Leontina Elena Filipiuc, Mariana Pinteala, Cristina Mariana Uritu, Bogdan Ionel Tamba

**Affiliations:** 1 Centre of Advanced Research in Bionanoconjugates and Biopolymers, “Petru Poni” Institute of Macromolecular Chemistry, 700487 Iasi, Romania; silviu.filipiuc@umfiasi.ro (S.I.F.); natalia.simionescu@icmpp.ro (N.S.); adina.coroaba@icmpp.ro (A.C.); nmarangoci@icmpp.ro (N.L.M.); pinteala@icmpp.ro (M.P.); 2 Advanced Center for Research and Development in Experimental Medicine “Prof. Ostin C. Mungiu”, “Grigore T. Popa” University of Medicine and Pharmacy, 700115 Iasi, Romania; gabriela-dumitrita.stanciu@umfiasi.ro (G.D.S.); leontina.filipiuc@umfiasi.ro (L.E.F.); bogdan.tamba@umfiasi.ro (B.I.T.)

**Keywords:** Rhein, fluorescence imaging, liposomal Rhein, Rhein biodistribution, Rhein cytotoxicity

## Abstract

**Objectives:** This work aimed to develop and investigate liposomes incorporating Rhein (Lip-Rh) into the liposomal membrane to enhance the compound’s water solubility and oral bioavailability. **Methods:** Liposomes were produced by the thin lipid film technique, with a phosphatidylcholine-to-cholesterol molar ratio of 5:1, dissolved in chloroform and methanol, and thereafter hydrated with ultrapure water and subjected to sonication. The resultant liposomes were studied from a physicochemical perspective using DLS, zeta potential, STEM, UV–Vis, and fluorescence spectroscopies, while oral bioavailability was assessed by fluorescence imaging. Additionally, cell viability assays were performed on tumour cells (MCF-7) in comparison to normal cells (HGFs). **Results:** The resultant nanoparticles exhibited relatively uniform sizes and narrow size distribution. In vivo fluorescence imaging studies performed on Wistar rats demonstrated significantly enhanced oral bioavailability for Lip-Rh, with rapid absorption into the bloodstream observed one hour after administration, in contrast to the free compound dissolved in vegetable oil. Cell viability assays demonstrated higher cytotoxicity of Lip-Rh towards MCF-7 cells compared to HGF cells, highlighting the selective therapeutic potential of the product. Moreover, we determined that the optimal dose of Rhein per kilogram of body weight, when encapsulated in liposomes, is approximately 2.5 times less than when Rhein is delivered in its unencapsulated form. **Conclusions:** Lip-Rh is a promising candidate for oncological treatments, presenting three key advantages: increased cytotoxicity towards tumour cells, protection of normal tissues, and the practicality of oral delivery. Additional investigation is required to explore its application in anticancer therapy, whether as monotherapy or as a complementary treatment.

## 1. Introduction

Rhein, or 4,5-dihydroxyanthraquinone-2-carboxylic acid, is a lipophilic anthraquinone sourced mainly from plant species such as *Rheum palmatum*, *Rheum officinale*, or *Cassia tora*. The compound has historically been used in Chinese medicine for its anti-inflammatory [1], antibacterial [2], and antidiabetic effects [3]. Modern pharmacological research has broadened its applications across multiple biological activities, positioning it as a viable candidate for numerous therapeutic uses, including as an anticancer agent [4]. Antitumour properties have been demonstrated on various cancer types, including breast [5], cervical [6], colon [7], lung [8], and pancreatic cancers [9]. The anticancer effect is mediated through the modulation of several pathways, such as the signal transducer and activator of transcription 3 (STAT3). Furthermore, numerous studies have demonstrated its ability to induce apoptosis, inhibit proliferation, and suppress angiogenesis [9]. The anti-inflammatory effects of Rhein are linked to its capacity to inhibit the NF-κB signalling pathway, which reduces inflammation and mitigates tissue damage. This phenomenon has been observed in diseases such as rheumatoid arthritis [10] and osteoarthritis [11]. Rhein shows promise as an antidiabetic agent by regulating blood glucose levels and decreasing lipid concentrations via the modulation of the PPARγ and AMPK signalling pathways [4,12,13]. It also demonstrates significant antimicrobial and antiviral properties, exhibiting broad-spectrum activity, including efficacy against bacterial strains resistant to conventional antibiotics [14,15].

Rhein has drawn significant interest from researchers, leading to extensive studies on its mechanism of action, particularly in its role as an antitumour agent. Rhein has shown synergistic effects with EGFR inhibitors, such as erlotinib, in the context of pancreatic cancer. The mechanism involves the inhibition of STAT3 phosphorylation, which is crucial in the advancement of pancreatic tumours. Studies on xenograft models indicates a notable decrease in tumour growth and the promotion of apoptosis [9]. Similarly, Rhein induced apoptosis in breast cancer cells through the activation of caspases and the inhibition of NF-κB, leading to a reduction in cell proliferation. These effects were demonstrated both in vitro and in vivo, supporting its application as an additional therapeutic agent [4,5]. In patients with colorectal cancer, Rhein’s anti-inflammatory properties, along with modulation of the TGF-β1/α-SMA axis, played a role in decreasing fibrosis linked to colorectal tumours. Rhein inhibits the progression of metastasis and enhances the efficacy of standard chemotherapy treatments [4,16]. It inhibited the PI3K/Akt pathway in lung cancer tumour cells, leading to reduced cell proliferation and the induction of apoptosis. Preclinical studies suggest its antitumour efficacy when used in combination with other targeted therapies [4,17]. In liver cancer, Rhein modulates gene expression related to liver regeneration and prevents fibrotic accumulation in hepatocellular carcinoma. Additionally, it reduces oxidative stress, contributing to the protection of normal cells [9].

Despite its promising pharmacological profile, Rhein poses several challenges, such as low water solubility, poor oral bioavailability, and a significant risk for hepatotoxicity. Recent advancements in the formulation of this compound include liposomal encapsulation, enhancing its solubility and therapeutic efficacy [18].

Innovative drug delivery systems, such as liposomes and nanoparticles [19,20,21], have been investigated to address the limitations of Rhein. These systems improve its stability, bioavailability, and targeted delivery. Combination therapy with other agents has also demonstrated synergistic effects, especially in the context of cancer treatment [4,17].

Liposomes are nanometric vesicular structures formed through the self-assembly of phospholipids in an aqueous environment. These systems are widely used for drug delivery due to their high biocompatibility, ability to encapsulate active compounds, and structural flexibility. One of the most beneficial features of the liposomes is their ability to enable the direct transfer of active compounds into cells via cellular internalization through various mechanisms, including lipid exchange, surface interactions, membrane fusion, endocytosis, and pinocytosis [22]. These nanocarriers, as they are also called, are used in various domains including nanomedicine, the cosmetics industry, and biotechnology [17,18,23]. Liposomes offer several advantages, including biocompatibility and safety, due to their phospholipid structures resembling cell membranes. This similarity significantly reduces side effects [17,18], facilitating controlled delivery and sustained release of active substances, while also protecting them from enzymatic or chemical degradation [18].

Liposomes can be functionalized with specific molecules to enhance delivery efficiency to targeted sites, including tumour cells [17,24]. They show increased versatility, allowing the encapsulation of both hydrophobic and hydrophilic compounds, and can be used in a wide range of applications [18].

Various techniques exist for the production of liposomes, each presenting distinct benefits and drawbacks. One method for obtaining liposomes is the thin lipid film method, which involves evaporating the organic solvent to form a thin phospholipid film, followed by hydration with an aqueous medium. It is a simple and frequently used method, but it produces liposomes of heterogeneous sizes [17,18]. Resizing methods, such as sonication, can effectively control the size of liposomes by applying ultrasound to the suspension, thereby reducing their dimensions. However, this method can degrade the active compounds [17,25]. Another method to resize liposomes obtained through the thin lipid film technique involves the extrusion technique, in which the liposome suspension is forced through membranes of specific pore sizes to achieve uniform liposome dimensions. This membrane-passing technique is effective for producing liposomes with precise sizes [18]. A further approach to liposome production involves the use of supercritical fluids. This method produces liposomes that are smaller in size and more efficient at encapsulating active substances. This technique is regarded as eco-friendly; however, it requires specialized equipment [25], which is significantly more complex than that required for the thin lipid film technique. Microfluidics can also be used as a method for obtaining liposomes and involves using microfluidic channels to control liposome formation, allowing small-scale production of liposomes with high reproducibility [26].

Various types of liposomes can be produced through liposome preparation techniques. Small unilamellar vesicles (SUVs) are characterized by diameters less than 100 nm and are used for the delivery of drugs and genetic material [17,18]. Large unilamellar vesicles (LUVs) range in size from 100 to 1000 nm and serve the purpose of encapsulating high-molecular-weight compounds [18]. Multilamellar vesicles (MLVs) consist of multiple phospholipid layers and are applicable for controlled drug release [17,25]. Giant unilamellar vesicles (GUVs) are primarily used in research to investigate membrane interactions [27].

Liposomes can be administered through various routes, with each method presenting distinct advantages and challenges depending on the targeted pathology and the specific formulation [17,18]. Intravenous (IV) administration is a primary route for liposome delivery and is the preferred method in oncological therapies. This method maximizes bioavailability and facilitates the rapid and safe entry of liposomes into systemic circulation, leading to their accumulation in affected tissues via the enhanced permeability and retention (EPR) effect [17,28]. Intravenous administration offers maximum bioavailability; however, it requires specialized equipment and poses a risk of systemic toxicity associated with liposomes. Intramuscular (IM) administration is an alternative method that facilitates the prolonged release of the active substances. Liposomes are retained in muscle tissue and gradually released into circulation. This method is particularly employed in vaccine technology and for the administration of analgesic substances [29,30]. The oral administration of liposomes is both accessible and convenient; however, it poses significant challenges due to gastrointestinal degradation and limited absorption. Nevertheless, liposomal formulations can protect active compounds from digestive enzymes, facilitating intestinal absorption [25]. The inhalation administration of liposomes is applicable for pulmonary conditions, minimizing systemic toxicity [17,31]. Liposomes can be inhaled for treating pulmonary diseases such as cystic fibrosis, respiratory infections, and lung cancer [28]. Subcutaneous (SC) administration is an alternative for the sustained release of liposomes, which enter circulation via the lymphatic system. The small size of liposomes (80–90 nm) and the presence of PEG in their composition enhance their stability and facilitate efficient absorption [17].

Topical/transdermal administration allows liposomes to be used in topical formulations for delivering drugs into or through the skin (transdermal). This method is employed in dermatological treatments and the cosmetics industry for the administration of active substances [25,32].

The administration methods for liposomes are diverse and can be tailored to meet specific therapeutic needs. Ongoing developments in liposomal systems are expected to optimize their application in addressing complex medical conditions. In this context, oral administration via gavage is particularly significant in preclinical research for assessing the efficacy of drug delivery systems. This method provides several advantages in animal studies, such as precise dosing and reproducibility of results.

Research has focused on orally administered liposomes to enhance the bioavailability of compounds that exhibit limited gastrointestinal absorption. Oral administration of liposomes presents challenges, particularly in overcoming the gastrointestinal barrier, as liposomes must withstand enzymatic and chemical barriers in the gastrointestinal (GI) tract and adapt to the intestinal pH. Digestive and bile juices can destabilize the liposomal structure, leading to the premature release of active contents [33].

The size and composition of liposomes play a critical role in their absorption. Small liposomes (<200 nm) are the most efficient for intestinal absorption due to their ability to cross the epithelial barrier. Coating with polyethylene glycol (PEG) results in improved stability and enhanced gastrointestinal absorption [33]. Cholesterol stabilizes the liposomal structure, thereby improving its resistance to acidic pH.

This study focused on developing liposomes that encapsulate Rhein (Lip-Rh) within the liposomal membrane to address the compound’s poor water solubility. A preparation method was adopted to ensure the production of liposomes with precise dimensions and a narrow size distribution. Additionally, a multilamellar liposomal structure design was chosen to maintain the integrity of liposomes post-intestinal absorption, aiming to facilitate their application in oral administration. This study evaluates the product’s efficacy using viability tests on normal and pathological cell cultures, alongside in vivo assessments of bioavailability following oral administration. The bioavailability assessment was designed using in vivo fluorescence imaging studies, leveraging the fluorescent properties of the compound. The biodistribution of Lip-Rh was compared to that of free Rhein (Rh), which was dispersed in a vegetable oil, by scanning the animals at the same time intervals following oral administration.

## 2. Materials and Methods

### 2.1. Materials

Phosphatidylcholine, soy PC (95%), and L-α-phosphatidylcholine (95%) were acquired from, Avanti Research™, Birmingham, AL, USA; cholesterol, methanol for HPLC, chloroform, and almond oil from Prunus dulcis were purchased from Sigma-Aldrich, Darmstadt, Germany; Rhein was acquired from Biosynth Carbosynth, Compton, UK.

### 2.2. Preparation of Rhein-Loaded Liposomes (Lip-Rh)

**Scheme 1:**
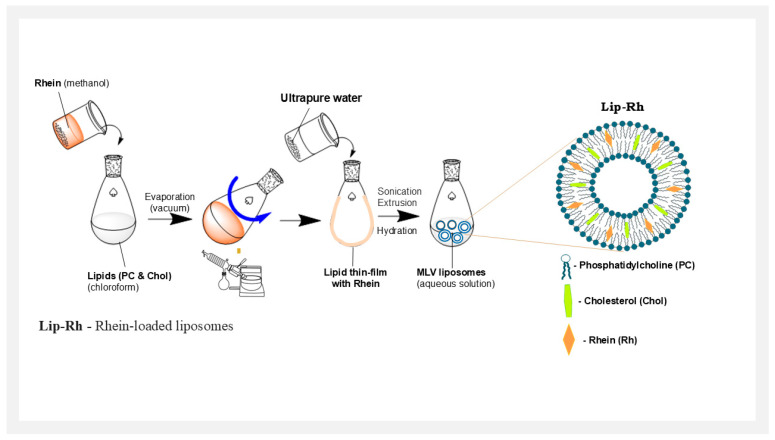
Workflow for the preparation of Rhein-loaded liposomes (Lip-Rh) using the thin-film hydration method.

The technique used to obtain the liposomes was the thin lipid film method [18,34], as shown in Scheme 1. The lipid film was prepared by solubilizing a mixture of phosphatidylcholine and cholesterol in a 5:1 molar ratio in chloroform. The required amount of Rhein was solubilized in methanol using a sonicator bath. The solution containing Rhein was transferred over the chloroform solution in which the lipids were solubilized, resulting in a methanol–chloroform ratio of 2.5:1 (*v*/*v*). This process in the end leads to a molar ratio of phosphatidylcholine–cholesterol–Rhein calculated as 5:1:0.125, reflecting the precise proportions of the components. The solution was subjected to evaporation using a rotary evaporator under vacuum for one hour at a speed ranging from 150 rpm to 250 rpm, and the pressure was gradually reduced by 100 mbar increments until reaching a minimum value of 38 mbar. Once the solvents were completely evaporated and the lipid film was observed, the flask was removed and placed in a vacuum drying oven at 10 mbar and a temperature of 23 °C, where it was left to dry for 24 h. The dried lipid film containing Rhein in the liposomal membrane was hydrated with ultrapure water and sonicated for 45 min in an ultrasound bath. To produce smaller and more uniformly sized liposomes, the extrusion method through membranes was applied, which facilitates the formation of smaller liposomes. This process utilized a Mini Extruder and PC Membranes 1.0 µm with pore sizes of 1 µm from Avanti Research™, Birmingham, AL, USA.

### 2.3. Liposomes Characterization

#### 2.3.1. The Size and ζ-Potential Measurements

The particle size and ζ-potential measurements were performed using Zetasizer Pro from Malvern Panalytical, Malvern, United Kingdom, employing dynamic light scattering (DLS) and electrophoretic light scattering (ELS) techniques, respectively. To achieve the measurements, disposable folded capillary cells, model DTS1070, were used. A volume of 1000 µL of a fresh liposome solution containing Rhein was transferred into the analysis cell, ensuring that no air bubbles formed in the cell’s capillaries. The cell was then placed in the cell-holder and subjected to the analysis protocol. The working temperature was set at 25 °C, with an equilibration time of 20 s. Three measurements were conducted on each sample, with 100 accumulations per cycle. The results reported are the average value of the three individual measurements.

#### 2.3.2. UV–Vis Spectroscopy

The absorbance measurements were performed using a PerkinElmer Lambda 25 UV/Vis spectrometer, Springfield, IL, USA, a high-precision analytical instrument widely employed for spectroscopic analysis of liquid and solid samples. The samples were analysed over a wavelength range of 200 to 700 nm, with the slit width fixed at 1 nm. A broad spectrum of the compound was examined to identify maximum absorbance peaks, which were subsequently utilized to plot the calibration curves for Rhein. Rhein samples of known concentrations were prepared through serial dilutions in a 1:1 (*v*/*v*) methanol–water mixture, starting from a stock solution with a concentration of 1 mg/mL. The stock solution was prepared by dissolving 20 mg of Rhein in 10 mL of methanol, followed by the addition of 10 mL of ultrapure water. Aliquots of 750 μL were transferred into 10 mm optical path-length glass microcuvettes for analysis, using a blank reference solution comprising an equivalent volume of the solvent mixture. To obtain the calibration curve, the following concentrations of Rhein were selected: 0.0005, 0.0008, 0.001, 0.003, 0.004, 0.005, 0.008, and 0.01 mg/mL.

The calibration curve was calculated taking into account the absorbance peak at 430 nm. The linearity was proven only in the range 0.0005–0.008 mg/mL, for which the R2 was found to be 0.99. To determine the exact concentration of Rhein in Rhein-loaded liposomes (Lip-Rh), which is supposed to be 2 mg/mL, 20 µL of liposome solution (of unknown concentration) was diluted in 20 mL of methanol and water up to 40 mL, to achieve a methanol–water ratio of 1:1 and a concentration below 0.008 mg/mL to ensure the measurements fall within the reliable range of the calibration curve. The UV–Vis spectrum was then recorded for the diluted sample, and the absorbance was read at 430 nm; to calculate the exact concentration, this dilution was taken into account.

#### 2.3.3. Fluorescence Spectroscopy

The fluorescence measurements were performed using FluoroMax-4 equipment (Horiba Scientific, Kyoto, Japan) in measurement cells with an optical path of 1 cm. The fluorescence spectra of different concentration of Rhein were achieved for the purpose of plotting a calibration curve for the compound. The emission spectra were measured in the 451–857 nm range, for identical sample volumes of 3 mL, under excitation at 436 nm. The operating parameters were as follows: 4 nm excitation slit width, 4 nm emission slit width, 100 nm/min scan speed, ten scans per sample, and room temperature. The emission spectra of Rhein-containing samples were corrected for the background signals. The 1 mg/mL stock solution of Rhein was prepared in a 1:1 (*v*/*v*) methanol–water mixture, first dissolving the compound in pure methanol, followed by sonication for 30 min at room temperature. From the stock solution, the following concentrations were prepared through successive dilutions: 0.0005, 0.0008, 0.001, 0.003, 0.004, 0.005, 0.008, and 0.01 mg/mL. The calibration curve was constructed considering the fluorescence peak at 576 nm. Linearity was demonstrated mainly within the range of 0.0005–0.005 mg/mL, generating an R2 value of 0.99. To establish the actual concentration of Rhein in Rhein-loaded liposomes (Lip-Rh), presumed to be 2 mg/mL, 20 µL of the liposome solution (of unknown concentration) was diluted in 20 mL of methanol and water to a final volume of 40 mL, achieving a methanol to water ratio of 1:1 and a concentration below 0.005 mg/mL, thereby ensuring that the measurements remain within the reliable segment of the calibration curve. The fluorescence spectrum was then recorded for the diluted sample, and the fluorescence emission was measure at 576 nm; to calculate the exact concentration, this dilution was taken into account.

#### 2.3.4. Morphological Analysis by STEM

The morphology of the Rhein-loaded liposomes was examined with the help of a scanning electron microscope type Verios G4 UC (Thermo Fisher Scientific, Waltham, MA, USA) working in STEM mode at 30 kV, with a STEM 3+ detector (bright-field mode). The samples were prepared by depositing 20 μL of a 1:10 diluted Lip-Rh solution onto carbon-coated copper grids with a 300 mesh size.

### 2.4. Cell Culture Assay

The cytotoxicity of compounds was assessed using the CellTiter-Glo^®^ 2.0 Assay (Promega, Madison, WI, USA) according to the manufacturer’s instructions. Human gingival fibroblasts (HGFs) and breast adenocarcinoma cells (MCF-7, both from CLS Cell Lines Service GmbH, Eppelheim, Germany) were seeded separately into 96-well opaque white tissue culture-treated plates (50,000 cells/mL—HGFs; 100,000 cells/mL—MCF-7) in αMEM medium with 10% foetal bovine serum and 1% antibiotic-antimycotic (all from Gibco, Thermo Fisher Scientific, Waltham, MA, USA) and allowed to adhere overnight. The next day, cells were incubated with different concentrations of samples (25, 50, 75, and 100 µM for both free Rhein and Lip-Rh) for 72 h. Unloaded liposomes were prepared and diluted the same as Lip-Rh for cell culture experiments. After 72 h, CellTiter-Glo^®^ reagent was added, and luminescence was recorded using a FLUOstar^®^ Omega microplate reader (BMG LABTECH, Ortenberg, Germany). The experiments were carried out in triplicate, and treated cells’ viability was calculated as a percentage of untreated cells’ viability. The data were represented graphically as means ± standard deviations.

### 2.5. In Vivo Experiments

#### 2.5.1. Animals

This study received approval from the Ethical Committee of the “Grigore T. Popa” University of Medicine and Pharmacy, Iasi (approval no. 116/18.10.2021), and was conducted in compliance with European legislation (Directive 2010/63/EU) and Romanian law (Law no. 43/2014) concerning the protection of animals used for scientific purposes. Additionally, authorization was obtained from the National Sanitary Veterinary and Food Safety Authority (authorization no. 50/18.02.2022). The experiments utilized adult female Wistar rats (Cantacuzino Institute, Bucharest, Romania) with an average weight of approximately 300 g. The animals were housed in the animal facility of CEMEX, “Grigore T. Popa” University of Medicine and Pharmacy, Iasi, in individually ventilated cages (IVCs) under controlled environmental conditions: temperature 20 ± 4 °C, relative humidity 50 ± 5%, and a 12 h light/dark cycle. The bedding consisted of sawdust, and the rats had ad libitum access to standard rodent chow and water. All animals used in the study were monitored for 14 days following the imaging experiments, and they survived in a good healthy condition.

#### 2.5.2. Spectral Imaging Equipment

The imaging experiments on animals were performed on AMI HTX equipment for preclinical research, from Spectral Instruments Imaging, Tucson, AZ, USA, which may acquire images in fluorescence, bioluminescence, and X-ray mode. It allows the detection and quantification of optical signals from sources related to the operating mode. It is equipped with a high-performance CCD camera cooled to −90 °C, ensuring high sensitivity and reduced background noise during image acquisition. The equipment can be set to work at 10 excitation wavelengths between 430 and 745 nm and also at 10 emission wavelengths between 530 and 790 nm. Data analysis was performed with the help of Aura software, version 4.5.0, which allows equipment control and automated quantitative image analyses, facilitating rapid data interpretation.

#### 2.5.3. Study Protocol

To analyse the bioavailability of the obtained liposomes, we used the fluorescent properties of Rhein, which was measured after being administered to animals. The rats (Wistar strain) employed in the study were divided into 3 groups (G1, G2, and G3), each containing 6 animals. The control group (G1) consisted of animals that were not administered any substance, with only the natural fluorescence background being measured. The animals in the second group (G2) received 20 mg/kg of bodyweight Rhein solubilized in almond oil (2 mg/mL). The third group (G3) consisted of animals that received a liposome solution prepared to obtain the same concentration of 2 mg/mL of Rhein, included in the liposomal membrane. Following this protocol, an animal weighing 300 g received by gavage a volume of 3 mL of oily suspension or liposome solution, respectively, each presumably containing 6 mg of Rhein. The animals were subjected to fluorescence imaging examination under isoflurane anaesthesia, at a time interval of one hour after the administration of the fluorescent compounds. The parameters set for data acquisition were as follows: an excitation wavelength of 430 nm, an emission wavelength of 690 nm, Binning 2, excitation power 35, exposure time 1, FOV 25, Fstop 2, and object height = 3.

### 2.6. Statistical Analysis

The experiments were conducted in triplicate, and the results are presented as the mean ± standard deviation. Data analysis and graphical representations were performed using Microsoft Excel 2010 and OriginPro 2020b.

## 3. Results and Discussion

The primary advantage of liposomes lies in their ability to enable the direct transfer of active compounds into cells [22]. This capability is closely influenced by the lipid composition of the liposomal membrane, which can be tailored to mimic cellular membranes for enhanced interaction and stability. Considering that cell membranes predominantly consist of phosphatidylcholine (PC) and cholesterol (Chol; up to 40% content in red blood cells) [35], we opted for a cholesterol concentration of approximately 20%. This proportion was selected to optimize the drug entrapment within the liposomal bilayer due to its highly hydrophobic nature. Additionally, the incorporation of cholesterol is known to reduce membrane permeability and improve vesicle stability in serum, thereby potentially enhancing the efficiency of cellular internalization [36].

Liposomes in this study were prepared using the thin lipid film method, involving the solubilization of a PC and Chol mixture in a 5:1 molar ratio in chloroform. Rhein (in a 0.125 molar ratio) was solubilized in methanol and subsequently transferred to the lipid solution. After the complete evaporation of solvents and the formation of the lipid film, the flask was transferred to a vacuum drying oven set at 10 mbar and 23 °C, where it was allowed to dry for 24 h. The dried lipid film incorporating Rhein within the liposomal membrane was rehydrated using ultrapure water. Two resizing methods were employed—sonication alone and the combination of sonication with extrusion—in order to obtain liposomes with uniform sizes and narrow size distribution.

**Figure 1:**
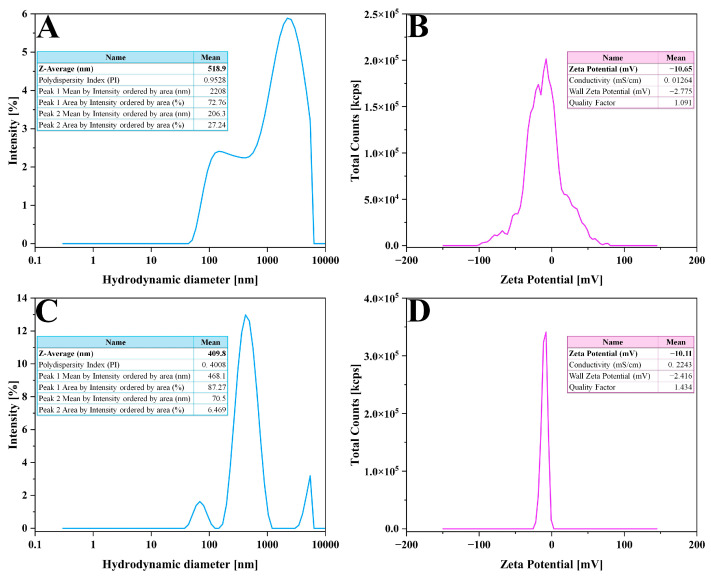
Size distribution profile of Lip-Rh liposomes in aqueous suspension, resized by sonication alone (**A**) and by sonication and extrusion (**C**), and the graph of ζ-potential distribution for the liposomes resized by the same methods: sonication alone (**B**) and sonication combined with extrusion (**D**). Extruded liposomes (mean diameter 409 nm), slightly smaller than non-extruded ones (mean diameter 518 nm), have a narrower size distribution and few exceeding 1000 nm. The ζ-potential profile is smoother in extruded liposomes compared to non-extruded ones, but with very similar values of 10.65 and 10.11 mV, respectively.

The liposome solutions were initially characterized using the dynamic light scattering (DLS) technique to determine their size and electrophoretic light scattering (ELS) to assess the ζ-potential, resulting in the data presented in Figure 1. The data from the DLS analysis provided a detailed overview of the size distribution and uniformity of the liposomes for both size-control methods: sonication alone and the combination of sonication with extrusion.

The hydrodynamic size of Lip-Rh prepared by sonication alone was measured to be 518.9 nm, as illustrated in Figure 1A. The results indicate that the liposomes obtained were relatively large, suggesting a population of multilamellar liposomes based on the preparation method employed. The polydispersity index (PDI), which quantifies the uniformity of particle size within the sample, was determined to be 0.9528. This value is slightly elevated yet remains within acceptable limits. This indicates a wide size distribution and significant heterogeneity, likely attributable to the preparation process, given that only the sonication method was employed. The figure illustrates two distinct peaks in the size distribution. The first peak corresponds to particles with an average size of 2208 nm, indicating a population of large particles (multilamellar liposomes). The peak area was 72.76%, indicating that this population predominated in light scattering intensity. The second peak corresponds to particles with an average size of 206.3 nm, indicating a smaller particle population, potentially consisting of unilamellar or oligolamellar liposomes. The proportion of this peak was 27.24%, signifying that this population constituted a minority within the sample.

Figure 1C illustrates the DLS data obtained for the liposomes resized using the combined method of sonication and extrusion through a 1 μm membrane. The liposomes showed an average hydrodynamic diameter of 409.8 nm, indicating the intensity-weighted mean size of the particles within the sample. The polydispersity index (PDI) of 0.4008 signifies a very good degree of size homogeneity in the sample. The size distribution indicated a dominant population with a mean size of 468.1 nm, representing 87% of the total intensity, suggesting that most liposomes are within this size range. Two additional populations were observed: one with a mean size of less than 100 nm and another with a mean size exceeding 1000 nm. Both populations contributed insignificant percentages, each representing approximately 6% of the total intensity.

Parameters including ζ-potential, conductivity, and charge distribution offered valuable insights into the properties of the system. The ζ-potential analysis was conducted to characterize the colloidal stability and behaviour of particles in suspension. Figure 1B,D depict the ζ-potential distribution of Lip-Rh, indicating the electrical charge at the boundary of the electric double layer surrounding the liposomes prepared through sonication and extrusion subsequent to sonication. The measurements showed average ζ-potential values of approximately −10.6 mV, consistent across both preparation methods. These results are in agreement with previously reported findings in the literature. Zhou and Raphael [37] have attributed such ζ-potential values to the presence of phosphatidylcholine (PC), which exhibits a negative charge at neutral pH. Additionally, this suggests that the ζ-potential is not significantly influenced by the liposome size. These values indicate moderate colloidal stability of the solution, accompanied by a potential risk of particle aggregation over time [19].

**Figure 2:**
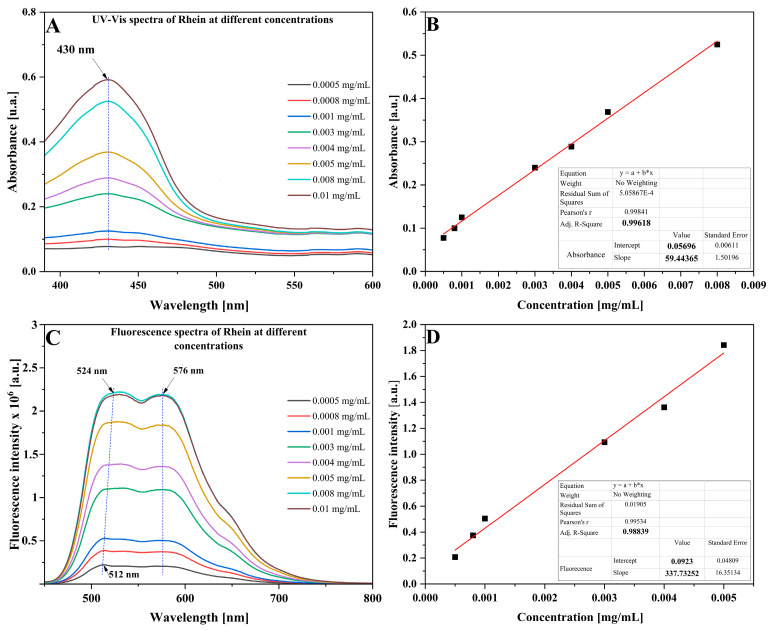
(**A**) UV–Vis spectra at different concentrations of Rhein; (**B**) UV–Vis calibration curve of Rhein solutions prepared in a mixture of water and methanol in equal proportions; the linearity was obtained in the range of 0.0005–0.008 mg/mL; (**C**) fluorescence spectra at different concentrations of Rhein; (**D**) fluorescence calibration curve of Rhein solutions prepared in a mixture of water and methanol in equal proportions; the linearity was obtained in the range of 0.0005–0.005 mg/mL.

Two calibration curves have been generated to evaluate the concentration of the bioactive molecule within the liposome membrane, depicting UV–Vis absorbance and fluorescence intensity as functions of Rhein concentration, as illustrated in Figure 2. Linear regression analysis was conducted using Origin 8.5 software to derive the mathematical equations for the two calibration curves. The linear ranges were determined to be 0.0005–0.008 mg/mL for the UV–Vis plot and 0.0005–0.005 mg/mL for the fluorescence plot. The selected ranges correspond to the highest R-squared values, with coefficients of determination calculated as 0.996 for UV–Vis and 0.988 for fluorescence, utilizing the maximum number of data points (concentrations).

**Figure 3:**
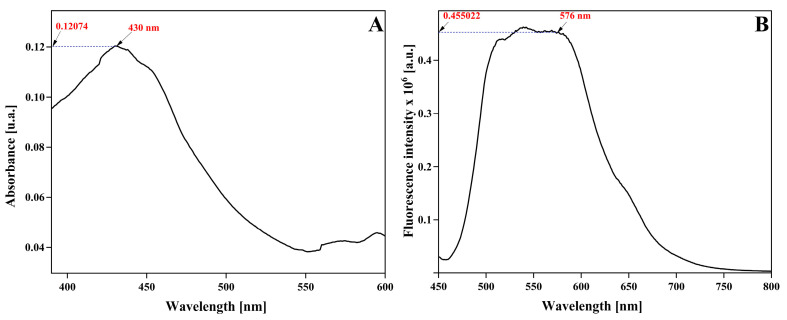
UV–Vis (**A**) and fluorescence (**B**) spectra of Lip-Rh solutions comprising an unknown concentration of Rhein. The representative peaks used in calibration curves at 430 and 576 nm are specified on the spectra and the corresponding read absorbance and fluorescence intensity.

The UV–Vis and fluorescence spectra of Rhein encapsulated in liposomes (of unknown concentration) closely resemble those of free Rhein, as illustrated in Figure 3. Both spectra maintain the characteristic maxima of absorbance and fluorescence emission at 430 nm and 576 nm, respectively, without any shifting of the specific peaks. The encapsulation process into the liposome membrane maintained the integrity of Rhein, with the surrounding lipid environment having a negligible impact on its absorbance or fluorescence intensity. However, some subtle differences can be observed in comparison to the spectra of the free compound. In the UV–Vis spectrum (Figure 3A), slight changes appear as shoulders, particularly with increased absorbance in the 500–600 nm range. In the fluorescence spectrum (Figure 3B), an additional peak emerges at 539 nm; however, it does not affect the primary fluorescence emission peak at 576 nm, which is essential for quantifying Rhein’s concentration via the calibration curve. These spectral changes suggest minor interactions between Rhein and the liposomal lipids, maintaining its optical properties or functionality.

The concentration of Rhein in the Lip-Rh product, derived from the calibration curves, was determined to be between 1.98 and 2.14 mg/mL across various batches of liposomes prepared under identical conditions. The concentrations were calculated using the corresponding mathematical equations: (1) A = 0.057 + 59.44·c for the UV–Vis plot and (2) FI = 0.092 + 337.73·c for the fluorescence plot, where A denotes absorbance, FI indicates the fluorescence intensity, and c represents the concentration.

The concentration of Rhein in liposomes determined by this method differs slightly from the estimated value of 2 mg/mL. This discrepancy can be attributed to human error, which may arise at multiple stages of the liposome synthesis process, including reagent weighing and the transfer of liposomes from the reaction flask to the storage vial. This aspect will be carefully considered during the preparation of liposome solutions intended for in vivo administration. Most notably, the concentration obtained for each liposome solution tested was almost identical, regardless of which calibration curve was applied. This detail enables us to conclude that either method can be reliably employed to determine the concentration of Rhein in a Lip-Rh solution of unknown concentration. Additionally, the fluorescence of the Lip-Rh product, which is not altered by the incorporation of Rhein into the liposomal membrane, makes it suitable for fluorescence imaging applications to study biodistribution in living organisms.

**Figure 4:**
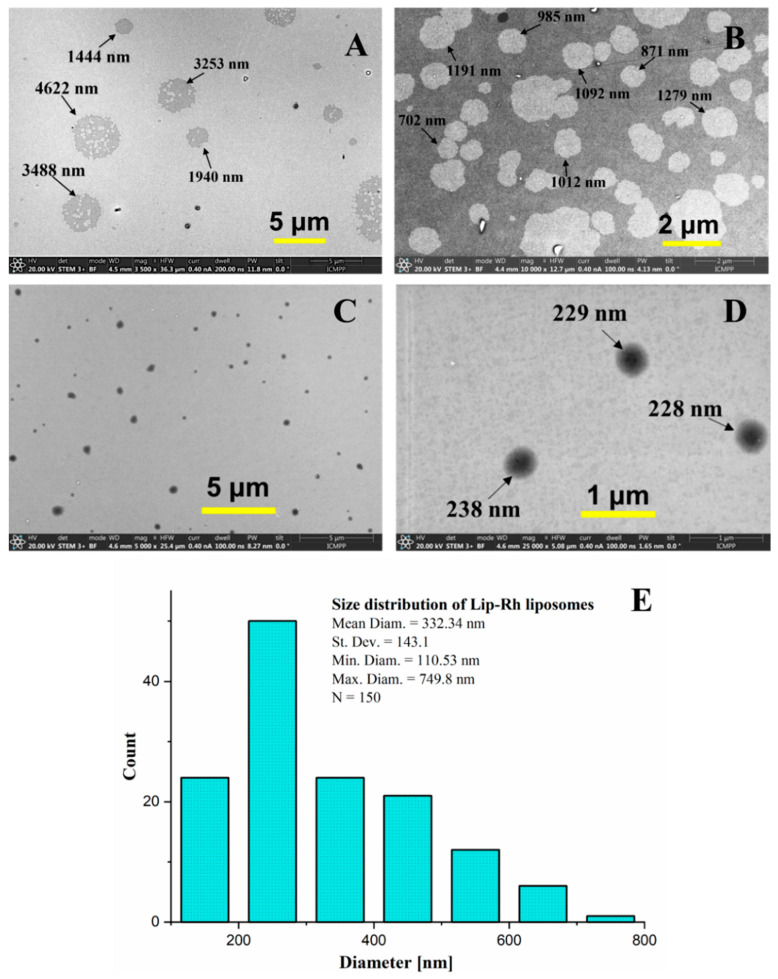
STEM micrographs of liposomes using as size-control methods: sonication alone (**A**,**B**) with the particles ranging between 100 and 5000 nm, and the combination of sonication with extrusion (**C**,**D**) providing a narrow size distribution between 100 and 800 nm and a mean diameter of 332 nm (**E**).

Figure 4 displays STEM micrographs illustrating the size distribution and morphology of Lip-Rh liposomes, which were prepared using two size-control methods: sonication alone and the combination of sonication with extrusion. The micrographs of liposomes obtained through sonication alone revealed a broad size distribution, with particle sizes ranging from 100 to 5000 nm (Figure 4A,B), highlighting the variability in liposome dimensions generated by this method.

In contrast, Figure 4C,D illustrate liposomes prepared using the combined method of sonication followed by extrusion, resulting in a markedly narrower size distribution. The liposomes demonstrate significantly improved uniformity, with a mean diameter of 332 nm, as depicted in the size distribution histogram (Figure 4,E). The statistical analysis of the particle sizes, based on approximately 150 particles measured from a limited set of STEM images, reveals a standard deviation of 143.1 nm, a minimum diameter of 110.53 nm, and a maximum diameter of 749.8 nm. These results highlight the efficacy of the extrusion step in improving the size uniformity of the liposome population, making this method suitable for applications that necessitate precise and consistent particle dimensions.

Analysing the STEM images of the formed liposomes, resized by extrusion, several relevant characteristics were observed, which are essential for evaluating the stability and efficacy of these types of liposomes as active substance delivery systems. It can be observed that the liposomes exhibit a typical bilayer structure, with a spherical shape and a well-defined structural uniformity (Figure 4D). It is also noteworthy that unextruded liposomes exhibit an approximately spherical morphology, accompanied by surface irregularities likely induced during the drying process of the samples deposited on electron microscopy grids [38]. These surface alterations are absent in small liposomes, indicating a lower membrane integrity and consequently a weaker structural stability of larger liposomes.

The predominant size of liposomes is between 200 and 300 nm, as shown in panel E; however, certain particles can reach diameters of up to 800 nm, which aligns with the utilization of a 1000 nm membrane during the extrusion process. The morphological and size attributes correspond properly with the optimal dimensions necessary for efficient delivery of active substances, especially in oral or intravenous administration. The encapsulation process has proven effective, as indicated by the successful integration of Rhein into the lipid matrix, with no observable separation or aggregation, suggesting that Rhein is uniformly distributed within the liposomal membrane. Importantly, no irregular inclusions or Rhein crystals were detected. The liposomes also demonstrate excellent structural stability, characterized by continuous, intact membranes with no visible cracks, ruptures, or significant pores. This level of stability is essential for ensuring controlled and efficient delivery of Rhein in vivo. Additionally, no significant interactions between the liposomes and the substrate used for imaging were observed, suggesting that the liposomal membrane maintains its structural integrity during the imaging process. These observations collectively highlight the potential of these liposomes as robust and effective delivery systems for active compounds.

The observed stability and structure of the analysed liposomes suggest their potential as an efficient tool for the controlled release of active substances, demonstrating high stability and effective incorporation of Rhein.

**Figure 5:**
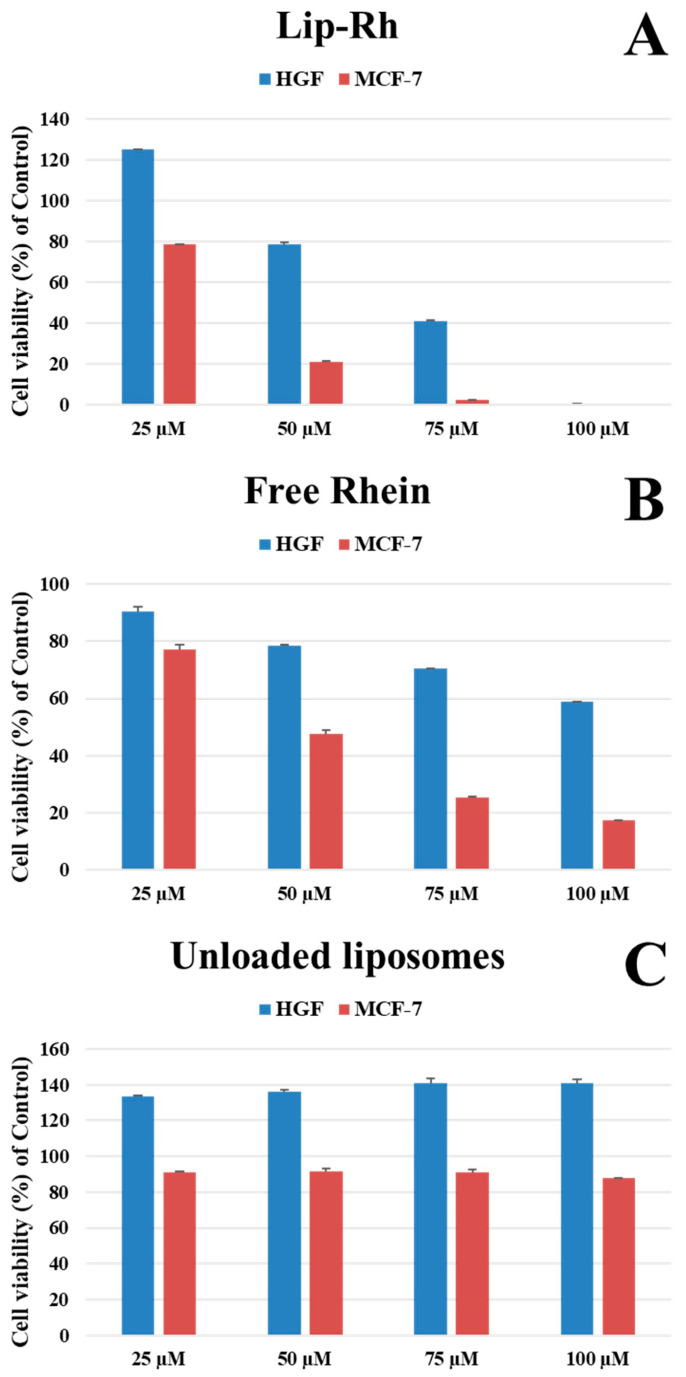
In vitro cytotoxicity assessment: differential impact of Lip-Rh (**A**), free Rhein (**B**), and unloaded liposomes (**C**) on normal (blue bar) and malignant (orange bar) cell lines, HGF and MCF-7 cells, respectively. At 50 µM, Rhein and Lip-Rh reduced MCF-7 viability, with a subtoxic effect on HGF cells (80%). Lip-Rh was more cytotoxic than free Rhein on MCF-7 (20% vs. 48% viability, respectively). Unloaded liposomes promoted fibroblast proliferation without affecting MCF-7 cells.

The biocompatibility of samples was assessed by viability tests on normal human fibroblasts (HGFs), while cytotoxicity was evaluated on the MCF-7 breast cancer cell line following a 72 h incubation period. We observed that at 50 µM concentration, Rhein and Lip-Rh were effective on MCF-7 cells while inducing a subtoxic effect on HGF cells, which survived in a proportion of 80% (Figure 5A,B). Lip-Rh demonstrated greater cytotoxicity towards MCF-7 cells than free Rhein, resulting in cell viabilities of 20% and 48%, respectively, while preserving the same 80% viability in normal fibroblasts. Unloaded liposomes significantly promoted the proliferation of normal fibroblasts, whereas the viability of MCF-7 cells remained unaffected (Figure 5C). Furthermore, malignant cells exhibited the highest sensitivity to all three treatments across all tested concentrations compared to normal cells.

The outcomes from in vitro testing were later adapted into in vivo experiments to establish a dose that is both effective and non-toxic. Therefore, to achieve a similar effect on tumour cells, Rhein encapsulated in liposomes may be administered at a concentration less than 50% of the dosage used in treatment with free Rhein. Based on the dosage of Rhein used in various studies concerning tumour treatment, specifically 50 mg/kg bodyweight [39,40], we propose that a dose of 20 mg/kg bodyweight of Rhein encapsulated in liposomes could be considered an optimal starting point for further investigation into its antitumour efficacy.

The in vivo studies were conducted on healthy female Wistar rats to evaluate the biodistribution and bioavailability of Lip-Rh after oral administration (see Materials and Methods, Section 2.5. In Vivo Experiments). The imaging investigations were performed using the AMI HTX system (Spectral Instruments Imaging), which enables detection and quantification of fluorescence within the animal body, providing spatial mapping of the fluorescent compound, Rhein. The AMI HTX equipment is widely used in studies related to disease progression in small animal models, therapeutic responses, and in vivo cellular migration. It is also suitable for early detection and monitoring of disease progression, providing high-quality images and essential quantitative data for biomedical research.

The administered dose of Rhein in animals was 20 mg/kg bw, as mentioned above, based on the results obtained from cell culture studies. The dose was identical in both liposomal and oily dispersion forms, comprising 6 mg of Rhein in a 3 mL administered volume of liposome solution and almond oil, respectively, for an animal weighing 300 g. Furthermore, due to the selection of oral administration and the necessity for a relatively large volume (3 mL), we decided to utilize a batch of multilamellar liposomes that were prepared exclusively by sonication, omitting the extrusion process. This decision relied on the assumption that multilamellar liposomes would remain largely intact after crossing the intestinal barrier. Only the outer membrane may be affected, thereby preserving liposomal structures with slightly reduced dimensions.

**Figure 6:**
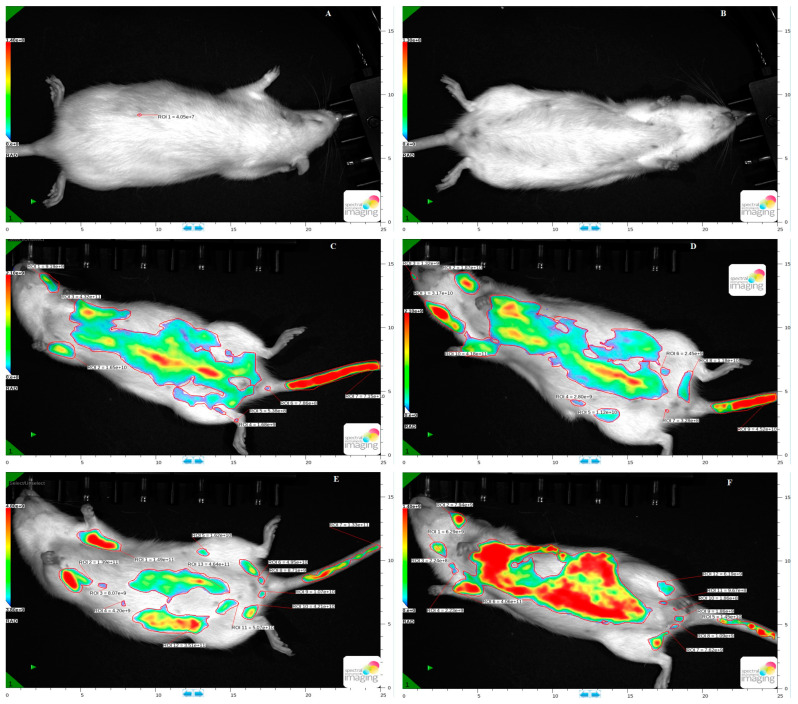
Qualitative in vivo fluorescence distribution of Rhein: control (**A**,**B**), liposomal delivery (**C**,**D**), and oil-based delivery (**E**,**F**). Lip-Rh showed strong signal accumulation in the abdominal region (likely liver and gut) and tail, indicating potential vascular retention. Rhein in oil displayed a more diffuse signal, with lower abdominal accumulation and non-uniform tail distribution, suggesting a slower systemic absorption.

Figure 6 illustrates the relevant images achieved from each animal group, which will be discussed below. The images of the control group animals indicate a low-intensity signal that is uniformly distributed across the surface of the animal’s body (Figure 6A,B). The signal may result from background noise or the inherent autofluorescence of tissues. No notable accumulations are detected in any region of interest, confirming the absence of any fluorescent compound.

In the group of animals receiving Rhein incorporated into the liposomal membrane, Lip-Rh, notable accumulations emitting a substantial intensity signal were observed in the abdominal region, likely the liver and gut. This indicates the presence of the compound in organs associated with the metabolism and elimination of administered substances (Figure 6C,D). An intense signal is observed in the tail, suggesting potential retention in the vascular or peripheral system. The absence of a signal in the head regions suggests that the administered compound does not penetrate the blood–brain barrier within a one-hour timeframe. Incorporating Rhein into the liposomal membrane appears to be effective in targeting delivery to organs, particularly those involved in metabolism. The more intense signal in the abdominal regions strongly suggests hepatic or renal accumulation, aligning with the functions of these organs in metabolism and elimination.

The signal for the group of animals receiving Rhein solubilized in oil is more diffuse than that for Rhein incorporated into liposomes (Figure 6E,F). Significant accumulations are observed in the abdominal region; however, these accumulations exhibit lower concentration. A non-uniform signal distribution is observed in the tail, suggesting systemic absorption via slow mechanisms, likely attributable to the oil-based formulation of Rhein. The signal in the head region, similar to the liposomal formulation, is weak, suggesting restricted distribution and indicating that the product does not penetrate the blood–brain barrier within one hour post-administration.

Rhein administered in oil exhibits a more uniform and diffuse distribution compared to the liposomal formulation, indicating slower absorption and reduced accumulation in metabolic organs. This approach may facilitate prolonged release; however, it is less targeted and likely to exhibit reduced effectiveness regarding antitumour activity, as indicated by cell culture results. The liposomal formulation exhibits improved delivery efficiency, characterized by a more concentrated distribution in metabolic organs, suggesting a potential enhancement in antitumour activity compared to an equivalent dose of Rhein in oil.

## 4. Conclusions

The research findings demonstrated that encapsulating Rhein within multilamellar liposome membranes significantly improved oral bioavailability, as evidenced by in vivo imaging studies conducted on Wistar rats. These studies revealed that the drug is rapidly absorbed into the bloodstream within one hour post-administration, unlike the free compound dissolved in vegetable oil. The liposomes prepared and characterized according to the described methods showed differentiated cellular viability in cell culture tests, exhibiting significantly higher cytotoxicity against tumour cells (MCF-7) in comparison to normal cells (HGFs). These results suggest that the liposomal Rhein possesses significant therapeutic potential in oncology, attributed to three primary advantages: elevated cytotoxicity towards tumour cells, protection of normal tissues, and the feasibility of oral administration. Additional studies are necessary to evaluate its effectiveness in anticancer treatment and to explore its potential application as a standalone therapy or as an adjunctive therapeutic agent.

## Data Availability

Data will be made available on request.
